# Supplementary figures and images for: Arctos: Community-driven innovations for managing natural and cultural history collections
Source: PLoS One. 2024 May 31;19(5):e0296478. doi: 10.1371/journal.pone.0296478 (PMC11142579; doi:10.1371/journal.pone.0296478)

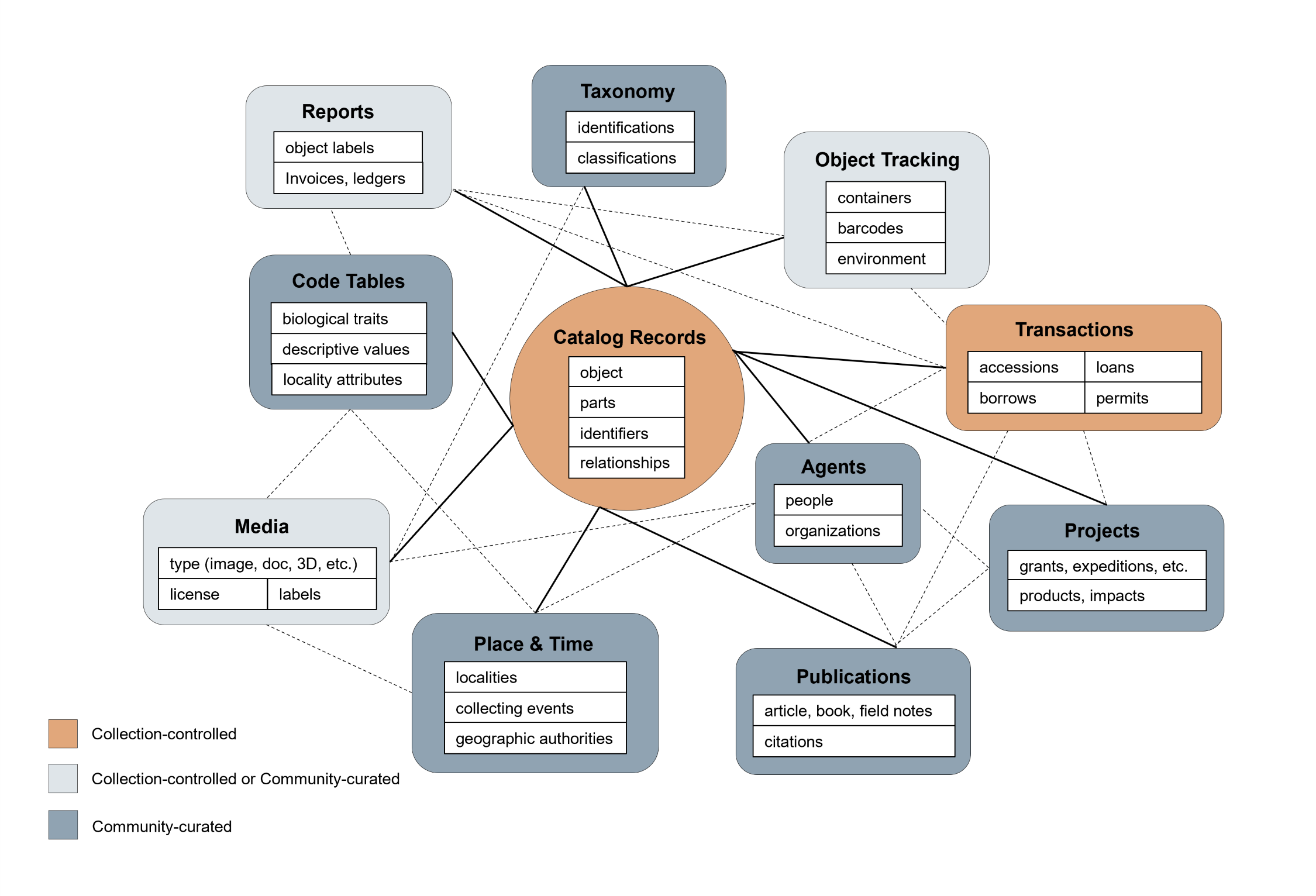

Supplement: S1 Fig — Schematic diagram highlighting data that are shared and curated across collections versus data that are controlled by individual collections. Some data can be collection-controlled or community-curated depending on the collection’s preference. Solid lines directly link data to catalog records, while dotted lines represent other relationships between tables. Certain shared data (e.g., localities) can be locked from editing by another user. Full details on all Arctos data tables and their structure are publicly available through the portal (https://arctos.database.museum/tblbrowse.cfm). (TIF) [file pone.0296478.s001.tif]

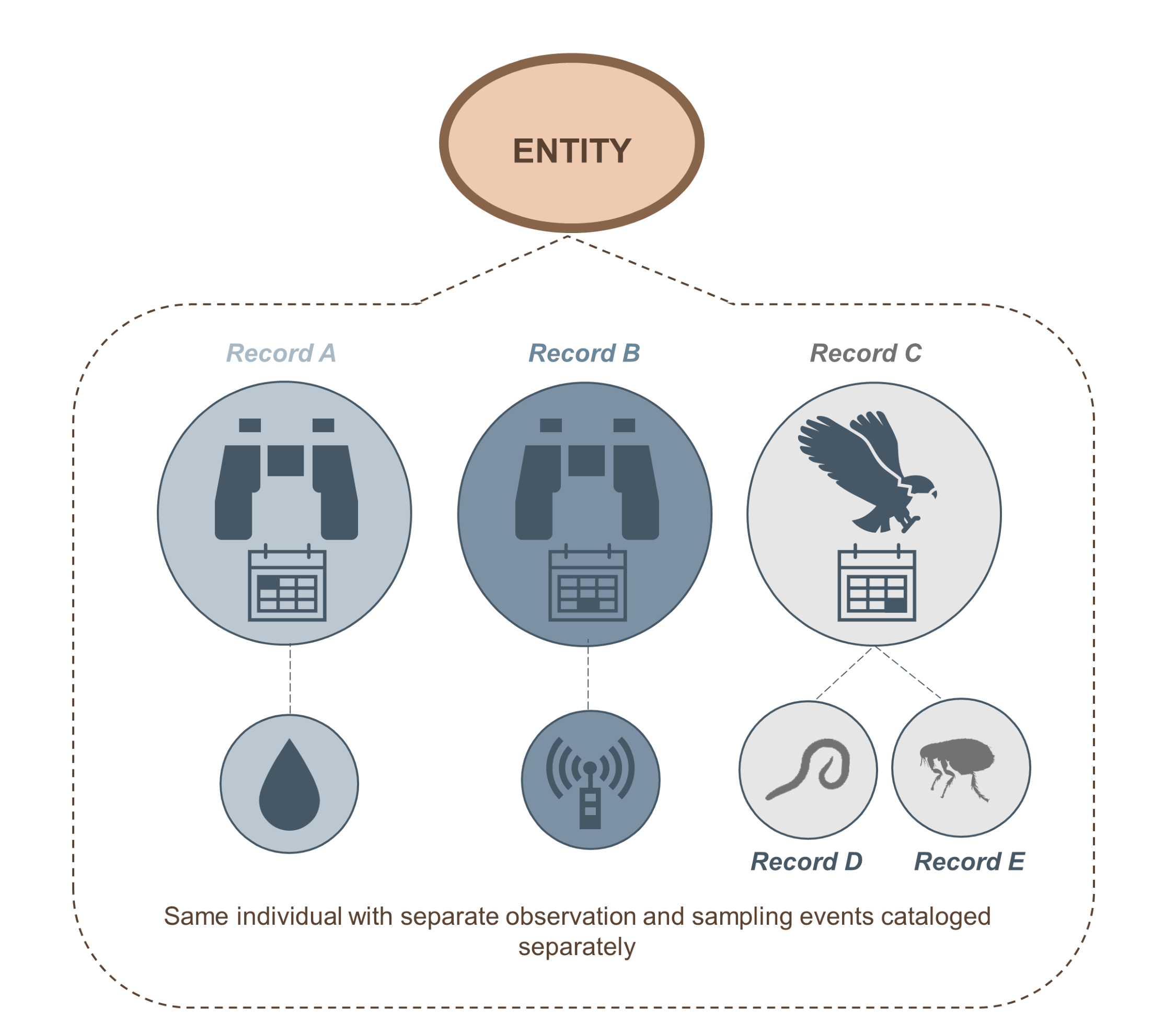

Supplement: S2 Fig — The Arctos Entity model links diverse catalog records to a single unifying record for increased discoverability. Here, Record A is a bird observation with an associated blood sample, Record B is a second observation of the same individual with associated radio telemetry data, Record C is the vouchered specimen, and Records D and E are endoparasites and ectoparasites, respectively, taken from the specimen. Three examples illustrate how the Entity record functions in Arctos to compile and unite multiple related occurrences or records of a single organism or collection object under one persistent identifier (Arctos base URL combined with the Darwin Core Triplet for the Entity record): (1) https://arctos.database.museum/guid/Arctos:Entity:16 links the cataloged blood sample of a Golden Eagle (Aquila chrysaetos) chick banded at its nest in 2014 (MVZ:Bird:193216) with data from a radio transmitter device that tracked the individual’s last known location to Mexico in 2017; that observation was cataloged in Arctos as MVZObs:Bird:4792. The coordinates for the original sampling locality are encumbered to protect the eagle nest. (2) A single endangered Mexican wolf (Canis lupus baileyi; https://arctos.database.museum/guid/Arctos:Entity:134) was monitored through a federal conservation program with regular blood sampling at different times in two zoos and the Wolf Management Facility, Sevilleta National Wildlife Refuge (MSB:Mamm:341613, MSB:Mamm:231704). Once moribund, the entire specimen was preserved and cataloged as MSB:Mamm:341614. (3) A captive Sumatran Orangutan (Pongo abelii; https://arctos.database.museum/guid/Arctos:Entity:204) in the Albuquerque BioPark Zoo has had at least 20 blood samples taken between 2011 and 2020 that are preserved in different ways and archived at the Museum of Southwestern Biology. (TIF) [file pone.0296478.s002.tif]
